# Relationship Between Species Diversity and Community Stability of Vegetation Patches in *Thymus mongolicus* Steppe, China

**DOI:** 10.3390/plants14081237

**Published:** 2025-04-18

**Authors:** Hui Wang, Mengyue Zheng, Honglin Xin, Bo Han, Hongju Jia, Fei Wu, Yunna Wu, Jing Ma, Yantao Song

**Affiliations:** 1 College of Environmental and Resource, Dalian Minzu University, 18 Liaohe West Road, Dalian 116600, China; 17735400425@163.com (H.W.);; 2 Bairin Right Banner Forestry and Grassland Bureau, Bairin Right Banner, Chifeng 025150, China

**Keywords:** *Thymus mongolicus* steppe, vegetation patches, species diversity, community stability, grassland degradation, diversity–stability relationship, ecosystem resilience

## Abstract

Grassland ecosystems play a crucial role in sustaining the stability of global ecosystem functions. However, the plant communities of grasslands exhibit spatially heterogeneous stability patterns such as vegetation patches influenced by human disturbances, herbivore activities, and climatic and topographic factors. This study investigated the vegetation dynamics in the *Thymus mongolicus* steppe in Bairin Right Banner, Inner Mongolia, analyzing the structural characteristics, species diversity, and community stability across six vegetation patches. Our findings revealed that patches dominated by grasses exhibited the highest values in coverage, height, density, and aboveground biomass. Besides, species diversity indices were highest in *Achnatherum splendens* patches and *Festuca litvinovii* patches, followed by *Thymus mongolicus* communities and *Leymus chinensis* patches, while the lowest diversity indices were observed in *Artemisia frigida* patches and *Convolvulus ammannii* patches. The order of community stability from high to low was *Leymus chinensis* patches, *Festuca litvinovii* patches, *Achnatherum splendens* patches, *Convolvulus ammannii* patches, *Artemisia frigida* patches, and *Thymus mongolicus* communities. Both the Patrick richness index and Margalef index showed a significant positive correlation with community stability (*p* < 0.05), indicating that plant communities with a higher species diversity tend to be more stable. These results emphasize the critical role of plant diversity in mediating community stability and contribute to the development of more effective grassland conservation and restoration strategies to maintain the health and sustainability of grassland ecosystems.

## 1. Introduction

Grassland ecosystems, constituting the largest terrestrial ecosystems globally, play a critically important role in maintaining the stability of global ecosystem functions [1,2,3]. Alarmingly, approximately 49% of global grasslands are currently degraded [4], a process strongly associated with landscape fragmentation. This degradation–fragmentation nexus manifests as a mosaic of vegetation patches characterized by distinct degradation states [5,6], progressively fragmenting intact plant communities into smaller, isolated remnants. Such fragmentation causes habitat loss and exacerbates ecological instability through edge effects and dispersal limitation [7], ultimately altering species diversity patterns and weakening ecosystem resilience [8].

Community stability, a comprehensive metric that reflects the structural integrity and functional continuity of an ecological community [9], is a crucial factor that determines a wide range of ecosystem services, from the preservation of biodiversity to the regulation of hydrological processes [10]. Fragmented grassland ecosystems are vulnerable to climatic extremes and anthropogenic pressures [11], necessitating urgent investigation into stability dynamics. Two principal theories explain how plant communities maintain stability: the diversity hypothesis posits that the stability of a plant community is closely related to its diversity [12], and the mass ratio hypothesis posits that the trait of the most dominant species, defined as the species with the highest relative abundance, serves as the primary driver of plant community stability [13,14,15].

The diversity–stability relationship remains a central debate in community ecology; while species diversity is widely regarded as a critical factor in determining plant community stability [16,17,18], the nature of this relationship remains elusive. A meta-analysis of 52 studies revealed that 69% demonstrated a positive correlation, 14% found a negative correlation, and 17% detected no obvious relationship [19]. Early theoretical frameworks proposed that species-rich communities exhibited enhanced stability through compensatory dynamics [20,21], with Elton’s foundational research asserting that “simple communities are more easily disrupted than richer ones” [21]. The perspective gained empirical support from Tilman’s [22] demonstration of diversity-mediated biomass stability and Naeem’s [20] functional redundancy hypothesis. Empirical evidence from grassland ecosystems, such as the Loess Plateau and northern Tibetan Plateau, further validated this positive relationship [23,24]. However, contemporary research reveals complex non-linear relationships, with some studies reporting destabilization effects at high diversity levels [25,26,27,28] or context-dependent correlations [29,30]. Furthermore, certain studies have argued against a simple linear relationship between these two variables, proposing instead a multivariate interaction [31]. Other researchers have contended that there is no significant correlation between species diversity and community stability [32]. The major drivers of stability and their relative importance in respective mechanisms remain unclear in different natural ecosystems [33,34]. This ongoing debate underscores the need for ecosystem-specific investigations, particularly in fragile grassland systems undergoing active degradation [35,36].

*Thymus mongolicus* steppe is a small shrub-dominated vegetation widely distributed in the Eurasian steppe region, playing an important role in the development of animal husbandry and soil and water conservation [37]. The Loess Hills in the Xilamulun River Basin is a representative region of the distribution of *Thymus mongolicus* steppe in China, and severe degradation has created a fragmented mosaic of vegetation patches in this region [38]. The fragmentation disrupts landscape connectivity, impeding vital ecological processes including seed dispersal and pollinator movement [39,40], thereby altering community composition and threatening ecosystem stability. Currently, research on the stability of this steppe is scarce and preliminary. Studying the fluctuations in diversity, stability, and their interrelationships among different types of vegetation patches is crucial for understanding ecosystem dynamics. This study aims to address whether the reduction in plant diversity in the *Thymus mongolicus* steppe mediated by patchy degradation will lead to a decline in community stability. Through a systematic analysis of patch characteristics, we specifically test two critical hypotheses: (1) Perennial graminoid-dominated patches maintain superior diversity and stability compared to other vegetation types; and (2) A positive correlation exists between plant diversity and community stability metrics in this degraded steppe system. This investigation provides mechanistic insights into the impacts of degradation while informing targeted restoration strategies for fragmented grassland ecosystems.

## 2. Materials and Methods

### 2.1. Study Area

**Figure 1:**
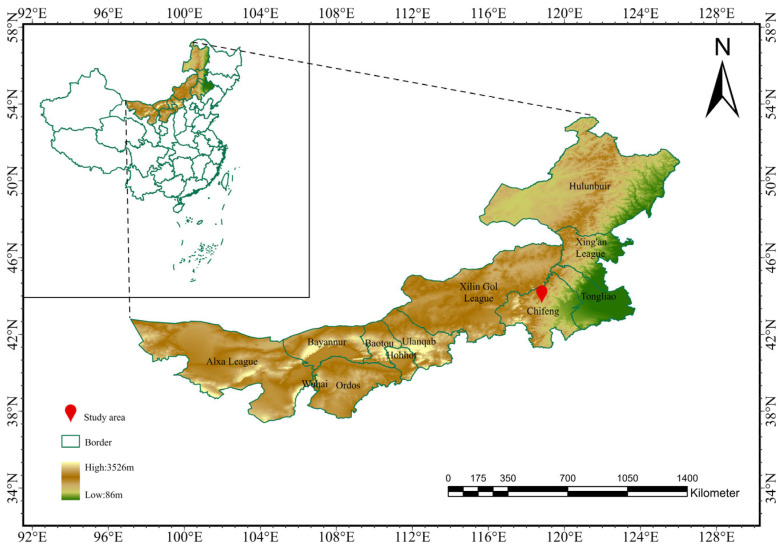
Overview map of the study area.

The study area was located in Bairin Right Banner, Chifeng City, within the Inner Mongolia Autonomous Region of China, spanning latitudes 43°12′ to 44°27′ N and longitudes 118°15′ to 120°05′ E (Figure 1). The topography features higher elevations in the northwest, reaching up to 1700 m, and lower elevations in the southeast, as low as 400 m, comprising three primary landform types: mountainous regions, hills, and plains. The region is characterized by a temperate semi-arid continental monsoon climate with four distinct seasons. Specifically, winters are cold and prolonged, while summers are short with concentrated rainfall. Spring and autumn show dramatic diurnal temperature fluctuations. The annual average temperature is approximately 4.9 °C, the annual average precipitation is about 358 mm, the annual average frost-free period is 125 days, and there are abundant sunlight resources, with the annual sunshine hours ranging from about 3000 to 3200 h [41]. The dominant vegetation types in the steppe are dry and semi-dry herbaceous plants and semi-shrubs, which are distributed in a patchy pattern.

### 2.2. Experimental Design and Sampling

**Figure 2:**
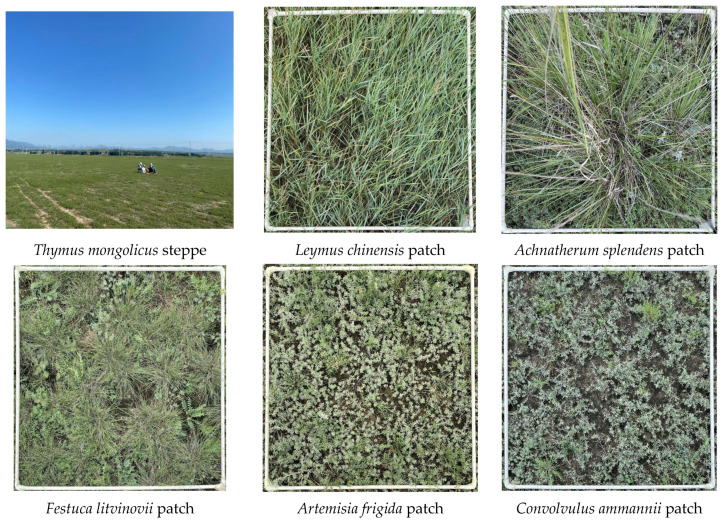
These are the sampling plots. (Credit: Yantao Song (photographer), Bairin Right Banner, Inner Mongolia, China. August 2024).

In mid-August of 2024, patches of plant communities dominated by *Leymus chinensis* (LCC), *Achnatherum splendens* (ASC), *Festuca litvinovii* (FLC), *Artemisia frigida* (AFC), and *Convolvulus ammannii* (CAC) were randomly selected in the *Thymus mongolicus* (TMC) steppe. Five 1 m × 1 m replicate plots were arranged in a randomization approach for each of the six vegetation types, and a total of 30 plots were established (Figure 2). Each sample plot was spaced at least 5 m apart. The quadrat method was used to investigate plant species and community characteristics. We recorded the names and numbers of the species occurring therein. When identifying species, we referred to the book *Common Plants Atlas of Grassland in Northeast China,* and we accessed http://www.iplant.cn/frps2019/ (accessed on 21 October 2024) to meticulously confirm the Latin names of the species. The natural vertical heights of three randomly selected individuals of the dominant species were measured using a tape measure, and the average value was calculated and taken as the community height. Density was determined by directly counting the number of clumps per square meter. Coverage was estimated visually. The aboveground biomass of each species was cut at ground level, dried at 75 °C for at least 48 h, and then weighed.

### 2.3. Data Calculations

#### 2.3.1. Community Species Importance Values

Important value (IV) [42] serves not merely to represent the distribution patterns of various species within a community but also to mirror their functional roles.

IV = (RC + RH + RD)/3 × 100%(1)

where RC is relative coverage, RH is relative height, and RD is relative density.

#### 2.3.2. Species Diversity Index

Diversity indexes are commonly employed to characterize the heterogeneity of plant communities [43]. This study quantified plant community diversity using five standard diversity metrics. Specifically, the Patrick richness index (R) and Margalef richness index (MA) were utilized to assess species richness. In addition, the Shannon–Wiener diversity index (H), Simpson dominance index (D), and Pielou evenness index (E) were adopted to evaluate other dimensions of biodiversity [43,44,45]. Their calculation formulas are as follows:

Patrick richness index (R):

R = S(2)

Margalef index (MA):

MA = (S − 1)/lnN(3)

Shannon−Wiener index (H):

(4) $$H=-\sum_{i=1}^{S}p_{i}lnp_{i}$$

Simpson index (D):

(5) $$D=1-\sum_{i=1}^{S}p_{i}^{2}$$

Pielou index (E):

E = H/lnS(6)

where S represents the number of species in the plant community, N indicates the total number of individuals of all species, and pi denotes the species important value.

#### 2.3.3. Plant Community Stability

This study employed the Godron contribution law method to evaluate the stability of plant communities. Following Zheng Yuanrun’s [46] and Lei Shilong’s [30] improved mathematical methods for Godron stability analysis, the reciprocal of the Euclidean distance was defined as the stability index to depict community stability. A higher value of this index indicates greater stability.

### 2.4. Data Analysis

Microsoft Excel was used for the initial organization and calculation of data. SPSS 27.0.1 software was employed for statistical analysis. Due to the relatively small sample size, the non-parametric Kruskal–Wallis test was applied to evaluate the differences in community characteristics and species diversity indices among different vegetation patches within the *Thymus mongolicus* steppe. Dunn’s test was used for comparison within the non-parametric Kruskal–Wallis test, and the significance values were adjusted by the Bonferroni correction method to control for Type I error inflation in multiple comparisons. All tests were conducted at the 0.05 significance level. The Spearman’s correlation coefficient was used to assess the monotonic correlation between community stability and diversity in different vegetation patches. ArcGIS Pro 3.1.5 was used to create an overview map of the study area. GraphPad Prism 10.1.2 was adopted to generate graphs of the species diversity index for vegetation patches, and Origin Pro 2022 was used to draw the remaining figures.

## 3. Results

### 3.1. Community Characteristics of Vegetation Patches

#### 3.1.1. Species Composition and Important Value

**Figure 3:**
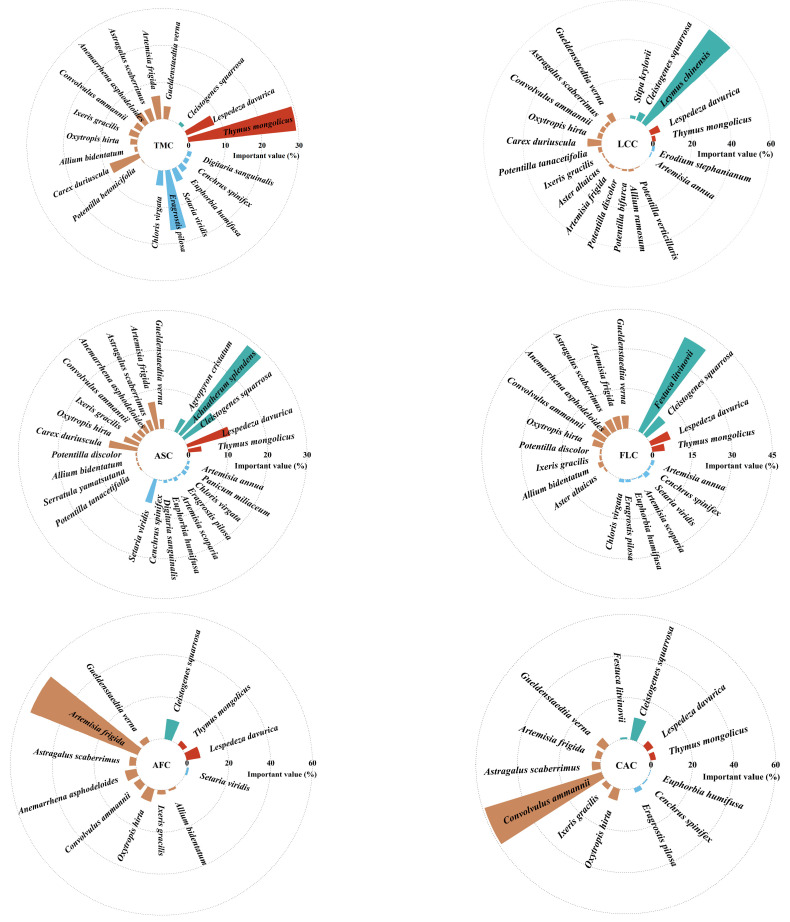
Species composition and important values of different vegetation patches in *Thymus mongolicus* steppe. TMC: *Thymus mongolicus* community; LCC: *Leymus chinensis* patches; ASC: *Achnatherum splendens* patches; FLC: *Festuca litvinovii* patches; AFC: *Artemisia frigida* patches; CAC: *Convolvulus ammannii* patches.

A total of 35 herbaceous plant species were identified in the *Thymus mongolicus* steppe. The numbers of species in the *Thymus mongolicus* community, *Leymus chinensis* patches, *Achnatherum splendens* patches, *Festuca litvinovii* patches, *Artemisia frigida* patches, and *Convolvulus ammannii* patches were 19, 20, 26, 21, 12, and 13, respectively. The dominant species demonstrated the highest important values across all communities. Specifically, the dominant species in the *Convolvulus ammannii* patches, *Leymus chinensis* patches, and *Artemisia frigida* patches had importance values of 59.0%, 58.9%, and 55.2%, respectively. In contrast, the dominant species in the *Festuca litvinovii* patches, *Thymus mongolicus* community, and *Achnatherum splendens* patches had importance values of 39.5%, 29.4%, and 28.5%, respectively (Figure 3).

#### 3.1.2. Quantitative Characteristics of Vegetation Patches

The community coverage of the *Leymus chinensis* patches was significantly greater than that of the *Convolvulus ammannii* patches (*p* < 0.05). The community height of the *Achnatherum splendens* patches was significantly greater than that of the *Thymus mongolicus* community, the *Convolvulus ammannii* patches, and the *Artemisia frigida* patches (*p* < 0.05). The community density of the *Artemisia frigida* patches, the *Leymus chinensis* patches, and the *Thymus mongolicus* community was significantly greater than that of the *Festuca litvinovii* patches (*p* < 0.05). The aboveground biomass of the *Achnatherum splendens* patches and *Leymus chinensis* patches was significantly greater than that of the *Convolvulus ammannii* patches (*p* < 0.05) (Table 1). 

### 3.2. Diversity Characteristics of Vegetation Patches

**Figure 4:**
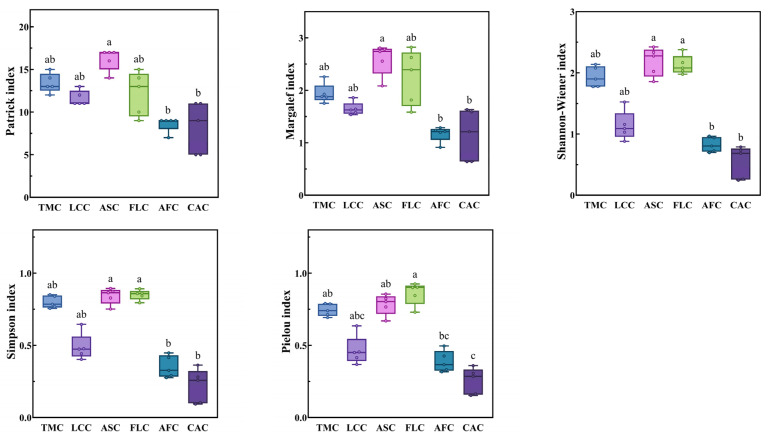
Species diversity index of different vegetation patches in *Thymus mongolicus* steppe. Different lowercase letters indicate significant differences among the vegetation patches (*p* < 0.05). TMC: *Thymus mongolicus* community; LCC: *Leymus chinensis* patches; ASC: *Achnatherum splendens* patches; FLC: *Festuca litvinovii* patches; AFC: *Artemisia frigida* patches; CAC: *Convolvulus ammannii* patches.

The Patrick index and Margalef index values of the *Achnatherum splendens* patches were significantly higher than those of the *Artemisia frigida* and *Convolvulus ammannii* patches (*p* < 0.05). In terms of the Shannon–Wiener index and Simpson index, the values for the *Achnatherum splendens* and *Festuca litvinovii* patches were both significantly higher than those of the *Artemisia frigida* and *Convolvulus ammannii* patches (*p* < 0.05). Similarly, for the Pielou index, the values of the *Festuca litvinovii* patches, *Achnatherum splendens* patches, and *Thymus mongolicus* community were significantly higher than those of the *Convolvulus ammannii* patches (*p* < 0.05) (Figure 4).

### 3.3. Stability Characteristics of Vegetation Patches

**Figure 5:**
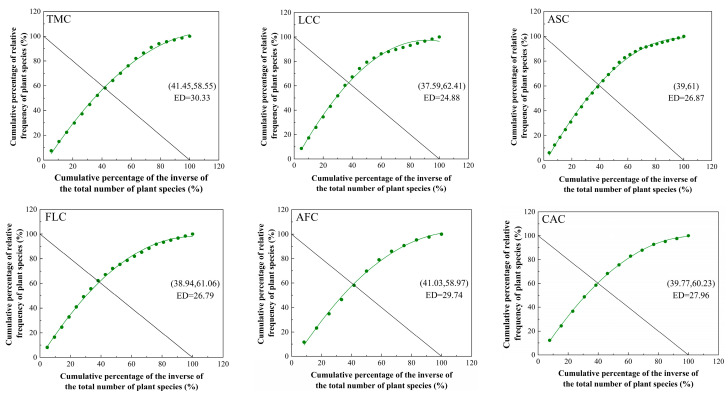
Simulation curves of M. Godron stability for different vegetation patch types in the *Thymus mongolicus* steppe. ED: Euclidean distance; TMC: *Thymus mongolicus* community; LCC: *Leymus chinensis* patches; ASC: *Achnatherum splendens* patches; FLC: *Festuca litvinovii* patches; AFC: *Artemisia frigida* patches; CAC: *Convolvulus ammannii* patches.

All of the intersection points of the patches demonstrated deviations from the theoretical stable point (20, 80). The *Leymus chinensis* patches had the highest stability index (24.88), followed sequentially by the *Festuca litvinovii* patches (26.79), the *Achnatherum splendens* patches (26.87), the *Convolvulus ammannii* patches (27.96), and the *Artemisia frigida* patches (29.74). Among all examined communities, the *Thymus mongolicus* community (30.33) exhibited the lowest stability among all examined communities (Figure 5).

### 3.4. Relationship Between Species Diversity and Community Stability Within Diverse Vegetation Patches

**Figure 6:**
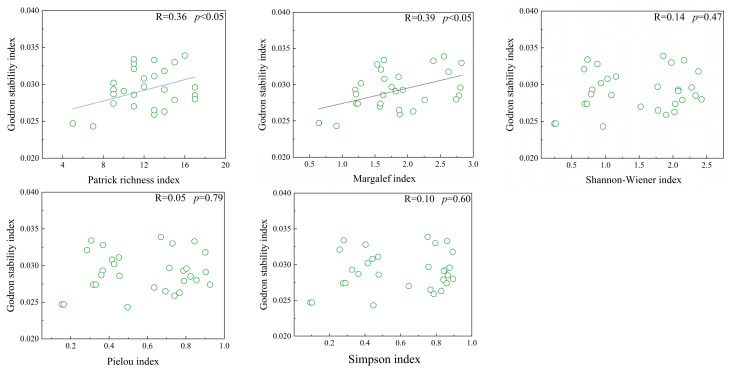
Linear regression analysis of species diversity and community stability in *Thymus mongolicus* steppe.

There were significant positive correlations between the stability index and both the Patrick index and Margalef index (*p* < 0.05). However, no significant correlations were found between the stability index and the Shannon–Wiener index, the Simpson index, or the Pielou index (Figure 6).

## 4. Discussion

### 4.1. Changes in the Community Characteristics of Different Vegetation Patches

Floristic investigation in the study area revealed a total of 35 herbaceous plant species which belonged to 12 families and which were distributed across 6 distinct plant community types. Among these species, the representatives of three dominant families were notable. The Poaceae family was represented by *Leymus chinensis*, *Achnatherum splendens*, and *Festuca litvinovii*; the Asteraceae family by *Artemisia frigida*; and the Convolvulaceae family by *Convolvulus ammannii*. These species exhibited obvious characteristics of concentrated distribution and formed a patchy distribution pattern. They played pivotal roles in maintaining the ecosystem integrity of the *Thymus mongolicus* steppe in Bairin Right Banner. The observed vegetation patchiness at the landscape scale can be primarily attributed to historical grazing patterns. Prolonged animal trampling, selective grazing, and heterogeneous distribution of animal excreta have led to significant spatial variations in soil moisture and nutrient availability [47]. These anthropogenic impacts are anticipated to persist during the initial phases of grassland restoration initiatives. Furthermore, the community characteristics of different vegetation patch types varied substantially, resulting from both intrinsic biological differences among plant species and extrinsic environmental factors, particularly soil microhabitat differentiation [11].

### 4.2. Changes in the Community Diversity of Different Vegetation Patches

Species diversity represents a fundamental property of plant communities and is widely recognized as a critical indicator for evaluating habitat quality, community structure, and species distribution patterns [48]. Research has indicated that dominant populations play vital regulatory roles in plant communities, significantly influencing both structural dynamics and functional processes [49,50]. The present investigation found substantial variations in species diversity across different plant communities. Notably, the *Achnatherum splendens* and *Festuca litvinovii* patches showed relatively high species diversity indices, while the *Artemisia frigida* and *Convolvulus ammannii* patches exhibited lower values. This is consistent with our first hypothesis. Furthermore, the analysis identified a significant negative correlation between species diversity indices and the important values of dominant species (*p* < 0.05), which is consistent with the findings of studies on *Stipa breviflora* grasslands in Inner Mongolia [51]. The regulation of species diversity is primarily mediated by interspecific competition and differential species responses to environmental heterogeneity. Since dominant species possess strong adaptability and tolerance within the region, their enhanced importance value intensifies their dominant effect, and this subsequently leads to the exclusion of other competing species, ultimately reducing community diversity [52]. This observed pattern supports the community self-thinning hypothesis, which suggests that, as resources become scarce, less competitive species are excluded, leading to a reduction in species diversity [53]. However, the important value of dominant species and the diversity of plant communities do not exhibit a simple linear relationship; instead, it is modulated by complex ecological interactions and feedback mechanisms. At regional scales, plant community diversity is simultaneously regulated by multiple interacting abiotic and biotic factors, such as topographic complexity, climatic variability, and soil physicochemical heterogeneity [54,55]. These environmental factors collectively establish a heterogeneous matrix of growth conditions that fundamentally influence community assembly processes, structural organization and ecosystem functioning. Particularly in arid and semi-arid regions, where environmental stresses are intensified, these factors exert disproportionately strong influences on the spatial patterning of plant diversity and the development of distinct habitat characteristics [56].

### 4.3. Relationship Between Species Diversity and Community Stability of Different Vegetation Patches

Plant community stability serves as a crucial ecological indicator that reflects not only spatial consistency but also temporal continuity in species distribution patterns [48], and it provides valuable insights into ecosystem health and resilience. It is fundamentally regulated by intricate networks of interspecific interactions, including both competitive and symbiotic relationships, which drive communities toward dynamic equilibrium states [49]. Empirical studies have shown that plant community stability is predominantly influenced by three key factors: interspecific competition intensity, environmental stress levels, and anthropogenic disturbance regimes [57]. Consistent with our first hypothesis, the *Leymus chinensis* patches exhibited the highest stability among all investigated communities in the *Thymus mongolicus* steppe, while the *Thymus mongolicus* community had the lowest stability. This diminished stability can be primarily attributed to the community being subjected to intensive grazing pressure. Livestock trampling and selective foraging behavior have substantially disrupted the structural integrity of the community and impaired its self-regulatory mechanisms. To comprehensively assess grazing impacts and establish reliable stability baselines, future research should incorporate comparative studies of *Thymus mongolicus* communities within long-term enclosures (with a minimum exclusion period of one year). Such research efforts will provide essential reference data for conservation and management strategies aimed at mitigating the negative effects of grazing on plant community stability in this ecosystem.

Species diversity, which encompasses both species richness and evenness, is indeed a robust metric for assessing community stability dynamics and is widely utilized to evaluate environmental impacts on ecosystem stability [58]. Consistent with our second hypothesis, there was a significant positive correlation between the species richness index and the community stability index in this study, which supports the view that increasing species diversity can promote the resilience and functional stability of the ecosystem (*p* < 0.05). These findings align with the ecological diversity–stability hypothesis and can be explained through multiple theoretical frameworks. The insurance hypothesis posits that augmented diversity heightens the probability of differential species responses to environmental changes and disturbances, providing a buffer against potential losses of species or functions. This is accomplished through an increase in functional redundancy, as species with similar ecological functions can substitute for one another when necessary [3,20,59]. Furthermore, the complementary effect theory posits that distinct species occupy specific ecological niches, and their functions exhibit mutual complementarity. As biodiversity increases, enhanced niche differentiation enables communities to access and utilize a broader range of resources, which leads to optimized resource allocation and increased stability [55]. These findings are consistent with previous research that highlights the crucial role of species diversity in regulating community stability [60,61]. However, it is important to note that diversity is not the sole driver of ecosystem stability. More precisely, ecosystem stability depends on the community’s capacity to maintain species or functional groups with diverse environmental response capabilities [53], which collectively enhance resilience and buffer against environmental fluctuations [62]. In addition to species diversity, other factors such as soil nutrient dynamics, environmental conditions, and external disturbance regimes also play a significant role in influencing grassland plant community stability [54]. Indeed, it is essential to recognize that the ecological mechanisms underlying the diversity–stability relationship are not static and can vary across different ecosystems and environmental contexts. A deeper understanding of these mechanisms facilitates the development of systematic grassland restoration strategies and the implementation of sustainable ecosystem management practices.

## 5. Conclusions

Plant community characteristics varied significantly among different patches in the *Thymus mongolicus* steppe (*p* < 0.05). Specifically, the *Leymus chinensis* patches, *Achnatherum splendens* patches, and *Festuca litvinovii* patches showed significantly better performance in coverage, height, density, and aboveground biomass compared to *Artemisia frigida* patches, *Thymus mongolicus* community, and *Convolvulus ammannii* patches. Diversity indices also differed among patches (*p* < 0.05), with the *Achnatherum splendens* patches, *Festuca litvinovii* patches, and *Thymus mongolicus* community displaying higher diversity index values than the *Leymus chinensis* patches, *Artemisia frigida* patches, and *Convolvulus ammannii* patches. A positive correlation between community stability and diversity was observed, indicating that species-rich communities tend to have greater stability. To effectively maintain species diversity and community stability in the *Thymus mongolicus* steppe of Bairin Right Banner, it is essential to comprehensively consider the compositional features, structural attributes, and influencing factors of plant communities and to develop management strategies tailored to site-specific habitat conditions.

## Figures and Tables

**Table 1:** Community characteristics of different vegetation patches in *Thymus mongolicus* steppe.

Community Index	Vegetation Patch Types M (P_25_, P_75_), n = 5						H	*p*
	TMC	LCC	ASC	FLC	AFC	CAC		
Coverage (%)	70 ab (60, 85)	92 a (90, 96)	92 ab (80, 95)	90 ab (80, 94)	92 ab (75, 94)	60 b (55, 75)	14.53	0.013
Dominant Species Height (cm)	5.8 b (4.8, 8.0)	46.5 ab (44.3, 50.3)	129.2 a (105.9, 176.2)	19.4 ab (15.9, 21.2)	5.3 b (3.3, 16.5)	5.5 b (4.7, 6.9)	24.55	<0.001
Density (plants/m^2^)	594 a (511, 735)	635 a (522, 743)	344 ab (308, 433)	143 b (142, 154)	721 a (571, 761)	510 ab (477, 637)	19.81	0.001
Aboveground Biomass (g/m^2^)	100.7 bc (96.5, 151.5)	320.5 ab (267.8, 329.9)	545.7 a (333.2, 558.4)	162.2 abc (152.7, 249.4)	217.2 abc (194.5, 360.4)	120.3 c (97.3, 124.1)	23.98	<0.001

Note: Different lowercase letters indicate significant differences among the vegetation patches (*p* < 0.05). M: median; P_25_: the 25th percentile; P_75_: the 75th percentile; TMC: *Thymus mongolicus* community; LCC: *Leymus chinensis* patches; ASC: *Achnatherum splendens* patches; FLC: *Festuca litvinovii* patches; AFC: *Artemisia frigida* patches; CAC: *Convolvulus ammannii* patches. H represents the test statistic in the Kruskal–Wallis test.

## Data Availability

The data presented in this study are available on request from the corresponding author. The data are not publicly available due to privacy.

## Abbreviations

The following abbreviations are used in this manuscript:

LCC	*Leymus chinensis* community
ASC	*Achnatherum splendens* community
FLC	*Festuca litvinovii* community
AFC	*Artemisia frigida* community
CAC	*Convolvulus ammannii* community
TMC	*Thymus mongolicus* community
